# Case Report: Long-term observations from the tacrolimus weaning randomized clinical trial depicts the challenging aspects for determination of low-immunological risk patients

**DOI:** 10.3389/fimmu.2022.1021481

**Published:** 2022-11-28

**Authors:** Christophe Masset, Jacques Dantal, Jean-Paul Soulillou, Alexandre Walencik, Florent Delbos, Sophie Brouard, Magali Giral, the Nantes DIVAT Consortium, Gilles Blancho

**Affiliations:** ^1^ Institut de Transplantation Urologie Néphrologie (ITUN), Centre Hospitalo-Universitaire (CHU) Nantes, Nantes, France; ^2^ Nantes Université, INSERM, Center for Research in Transplantation and Translational Immunology, UMR 1064, Nantes, France; ^3^ Laboratoire d’immunologie et HLA Etablissement Français du Sang, Nantes, France

**Keywords:** kidney transplantation, calcineurin inhibitor withdrawal, allograft rejection, donor specific antibodies, case report

## Abstract

Whilst calcineurin inhibitors (CNI) are the cornerstone of immunosuppressive maintenance therapy in kidney transplantation, several studies have investigated the safety of CNI withdrawal in order to avoid their numerous side effects. In this context, we performed several years ago a clinical randomized trial evaluating CNI weaning in stable kidney transplant recipients without anti-HLA immunization. The trial was interrupted prematurely due to a high number of *de novo* DSA (dnDSA) and biopsy proven acute rejection (BPAR) in patients who underwent tacrolimus weaning, resulting in treatment for rejection and resumption of tacrolimus. We report here the long-term outcomes of patients included in this clinical trial. Ten years after randomization, all patients are alive with a functional allograft. They all receive tacrolimus therapy except one with recurrent cutaneous neoplasia issues. Long-term eGFR was comparable between patients of the two randomized groups (46.4 ml/min vs 42.8 ml/min). All dnDSA that occurred during the study period became non-detectable and all rejections episodes were reversed. The retrospective assessment of HLA DQ single molecule epitope mismatching determined that a majority of patients who developed dnDSA after tacrolimus withdrawal would have been considered at high immunological risk. Minimization of immunosuppression remains a challenging objective, mainly because of the issues to properly select very low immunological risk patients. Valuable improvements have been made the last decade regarding evaluation of the allograft rejection notably through the determination of numerous at-risk biomarkers. However, even if the impact of such tools still need to be clarify in clinical routine, they may permit an improvement in patients’ selection for immunosuppression minimization without increasing the risk of allograft rejection.

## Introduction

In order to suppress the alloreactive immune system and thus avoid allograft rejection, a maintenance immunosuppressive therapy is required in kidney transplantation (1). This treatment is mostly comprised of a triple therapy: a calcineurin inhibitor (CNI, mainly tacrolimus), an antiproliferative drug (Mycophenolate Mofetil – MMF – or Mycophenolic Acid – MPA) and oral steroids. Whilst this strategy led to a large increase in kidney allograft survival (from 50% in the early 1960’s to 95% in 2020 (2) at one year), many transplant physicians have evaluated immunosuppression reduction in order to reduce the short and long-term drugs side effects. Some studies have demonstrated the possibility of steroids withdrawal (3, 4) or antiproliferative drugs withdrawal (5, 6) in selected patients with a low immunological risk. However, CNI, which leads to numerous side effects (metabolic complications, neoplastic risk, nephrotoxicity) still remain the cornerstone of the maintenance therapy. For a long time now, many studies reported the alloimmune consequences of CNI withdrawal in kidney transplant recipients (7, 8). However, these initial reports mainly concerned non-adherent kidney transplant recipients (KTR) or unselected patients, whilst other evaluated outcomes of tacrolimus withdrawal in the particular setting of low immunological risk KTR. In 2015, Hricik et al. conducted such a randomized clinical trial which was interrupted prematurely because approximately 35% of patients in the CNI withdrawal group developed biopsy proven acute rejection (BPAR) and/or *de novo* donor specific antibodies (dnDSA) (9). Our team also evaluated the possibility of tacrolimus withdrawal in selected patients with a low-immunological risk in a randomized clinical trial (10) (*NCT01292525*). Similarly, the study was interrupted prematurely due to a high incidence of allograft rejection and dnDSA in patients for whom tacrolimus discontinuation was achieved.

We describe here the long-term follow-up of the patients included in our tacrolimus weaning randomized clinical trial and discuss the challenging aspects for determination of low-immunological risk KTR which could be eligible for a drastic reduction of their immunosuppressive therapy.

## Case description

In 2010, we conducted a randomized trial in clinically stable kidney transplant recipients > 4 years displaying no DSA (Mean Fluorescence Index – MFI threshold < 1000) nor biopsy proven rejection (at the time of inclusion), comparing a complete and progressive CNI weaning (NO TAC) versus a standard immunosuppressive therapy by tacrolimus and antimetabolites (TAC) (10). On the 1500 screened patients, only 10 were finally enrolled. Included patients signed the informed consent form and underwent a double-blind randomization (1:1) in order to receive either standard tacrolimus (TAC) or a placebo (NO TAC). No additional immunosuppressive drug was added in patients who underwent tacrolimus withdrawal, and particularly no added oral steroids. A Supervisory Committee was responsible for ensuring trial safety and could decide at any time to stop the study.

The mean age of the included patients was 46 years. At the time of inclusion, the mean time since kidney transplantation was 6 years with an estimated glomerular function (eGFR by CKD-EPI) of 62 ml/min/1.73m (2) without significant proteinuria. All of them were recipients of a first kidney transplant, with an average number of HLA antigen mismatches of 5.4. The initial disease was IgA nephropathy for 5/10 patients. Seven patients received an initial induction by Basiliximab, followed by a maintenance therapy which consisted of tacrolimus and mycophenolate mofetil (average dose of 1,375mg/day) at the time of the inclusion. None of them received steroids at the time of the inclusion. One patient experienced T-cell mediated rejection (TCMR) several years before inclusion, which was successfully treated and resolved; and none of the patients had a CMV reactivation. For patients in the NO TAC group, 3/5 completely interrupted the tacrolimus therapy, whereas 2/5 where still in the progressive decrease period at trial interruption.

## Diagnostic assessment

The trial was prematurely interrupted because of immunological events in the NO TAC group: 3/5 patients displayed significant *de novo* DSA (dnDSA) within 3 to 6 months post randomization with a Mean Fluorescence Index > 2000. Among patients in the NO TAC group, one presented active antibody mediated humoral rejection (ABMR), one patient a TCMR and one patient a mixed rejection (ABMR + TCMR). ABMR lesions were treated by plasma exchange, Rituximab, and intravenous immunoglobulins, while TCMR lesions were treated by steroids pulses; added to resumption of standard doses of tacrolimus in all cases. This strategy allowed a total reversion of acute histological rejection lesions in the control biopsies (performed in a mean time of 11 months after the BPAR), and a disappearance of the DSA for all patients after a mean period of 7.3 months.

**Figure 1:**
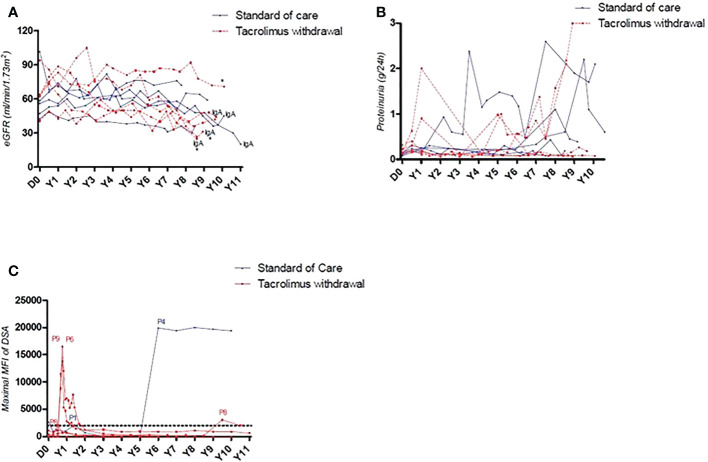
**(A)** Long-term evolution of allograft function of patients included in the CNI weaning protocol. All patients with a decrease > 50% of eGFR at long-term were those with IgA glomerulonephritis recurrence. **(B)** Long-term evolution of proteinuria of patients included in the CNI weaning protocol. **(C)** Representation of DSA’s maximal MFI. 3/5 patients who underwent tacrolimus withdrawal presented an early occurrence of de novo DSA with a MFI > 2000, quickly reversed after specific treatment. 2 patients developed a de novo DSA long term after the study, independently of their inclusion group.

About 10 years after the initial randomization, we analyzed the outcomes of these patients **(** **Table 1** **)**. All are alive with a functional graft. They all receive tacrolimus therapy except one with recurrent cutaneous neoplasia issues. Long-term eGFR was comparable between patients of the two randomized groups (46.4 ml/min vs 42.8 ml/min). A significant eGFR reduction (> 50%) was observed in 3 patients with IgA glomerulonephritis recurrence (2 in the NO TAC group and 1 in the TAC group, **Figures 1A, B**). Two patients were reported to have late adherence issues and thus developed another long-term dnDSA (6 and 9 years) after the study. Interestingly, all dnDSA that occurred during the study period following tacrolimus weaning remained non-detectable (MFI < 1000) at long-term follow-up (**Figure 1C**). Of note, 2 patients from the NO TAC group which presented a BPAR had a biopsy long after the study (9 years), and one presented chronic ABMR lesions despite the absence of DSA.

**Table 1:** Long term outcomes of patients included in the randomized clinical trial for CNI weaning.

Patient	Sex	TAC weaning*	Time from transplantation (years)	Time from randomization (years)	BPAR during study period	dnDSA during study period	Rejection treatment	Single Molecule DQ Eplet mismatch	Long term dnDSA	Observance	Glomerulonephritis recurrence	eGFR at inclusion	Long term eGFR	Chronic histological allo-immune lesions	Current immunosuppressive drugs
1	M	No	16	10	No	No	None	3	No	Appropriate	Ig A	59	20	No	Tac – MPA
2	M	No	17	10	No	No	None	16	No	Appropriate	Ig A	48	45	No	Tac – MPA
3	M	No	14	9	No	No	None	8	No	Appropriate	None	42	36	No	Tac – MPA
4	M	No	15	9	No	No	None	14	Yes	Inappropriate	None	47	59	No	Tac – MPA
5	F	No	12	8	No	No	None	0	No	Appropriate	None	91	72	No	Tac – MPA
6	F	Yes	16	10	Yes	Yes	Yes (1)	19	No	Appropriate	None	60	71	No	Tac – MPA
7	M	Yes	19	9	No	Yes	None	18	No	Appropriate	Ig A	65	42	No	Steroids
8	M	Yes	14	9	No	Yes	None	6	Yes	Inappropriate	None	49	44	No	Tac – MPA
9	M	Yes	14	9	Yes	Yes	Yes (2)	18	No	Appropriate	Ig A	69	31	Yes	Tac – MPA - Steroids
10	M	Yes	14	8	Yes	No	Yes (3)	9	No	Appropriate	Ig A	90	26	No	Tac – MPA

M, male; F, female; TAC, Tacrolimus; BPAR, Biopsy Proven Acute Rejection; dnDSA, de novo Donor Specific Antibody; eGFR, estimated Glomerular Function by MDRD in ml/min.

*: Because of early occurrence of immunologic complications, the trial was prematurely interrupted and standard maintenance therapy including CNI was resumed in all patients.

1: Steroids Pulses, Rituximab, Plasma Exchanges, IV-Ig.

2: Rituximab, Plasma Exchanges, IV-Ig.

3: Steroids Pulses.

Retrospectively, we assessed HLA epitope mismatching of the included patients using the HLAMatchmaker software (11). The average class I mismatch was 31 and the average class II mismatch was 25, none of the patients had thus an epitope mismatch load < 16. We then assessed specifically the single DQ molecule, recently described as a major factor in the risk of dnDSA occurrence (12), **Table 1**. In the NO TAC group 3/5 patients would have been considered at “high risk” with a single molecule DQ > 11; 2 of them indeed developed dnDSA following tacrolimus withdrawal. The two other patients would have been considered at “intermediate risk” with a single molecule DQ between 1 and 10; one of them developed dnDSA following tacrolimus withdrawal.

In the TAC group, 2/5 patients would have been considered at “high risk” with a single molecule DQ > 11, among whom one patient developed a dnDSA long term after the clinical trial. The 3 other patients would have been considered as “intermediate risk” for two of them, and “low risk” for one of them.

## Discussion

We reported the long-term outcomes of a small series of low immunological risk KTR who underwent CNI withdrawal in the context of a clinical trial that was interrupted prematurely due to a high rate of dnDSA and BPAR. Our results are consistent with the literature, as other studies have demonstrated a high risk of immunological complications following CNI withdrawal, even in KTR who were estimated with a low immunological risk (9, 13–15).

One important point in our study was the complete reversibility of dnDSA and histological rejection lesions (except for one patient who had lesions of cABMR several years later despite initial histological resolution of rejection lesions) following an early and intensive treatment of allograft rejection. Indeed, long term prognosis of ABMR is often depicted as poor mainly due to the persistence of dnDSA and allograft injuries despite specific treatment, even if some reported favorable outcomes in subclinical ABMR (16). The better prognosis of patients from our series may be linked to the timing of treatment instauration: because our patients were closely followed-up in the clinical trial setting, dnDSA were detected very early and biopsies performed as soon as possible in order to deliver adequate treatment. This resulted in a long-term survival of all patients who presented ABMR, with a disappearance of the DSA. The situation is quite different in a real-life setting where DSA are often detected after several months or years in - possibly non-adherent – patients. This may partially explain the observed treatment failures, due to the chronicity of alloimmune injury and thus support close long-term monitoring of DSA for patients at-risk.

Similar to other studies, our trial failed to demonstrate safety of a CNI withdrawal in selected KTR. Indeed, inclusion criterion of such clinical trials mainly relied on clinical and biological features that have been shown to increase the risk of rejection: recipient age (17), number of HLA mismatches (18) or presence of anti HLA antibodies (19). This reflects the difficulty of defining “low-immunological risk” patients, despite availability of risk stratification models (20). Consequently, establishment of patients’ immunological risk needs to be more accurate and thus should include other characteristics.

In this setting, the determination of HLA epitope mismatching have demonstrated a correlation with occurrence of dnDSA (21). These initial results were further confirmed by others, demonstrating that epitope mismatching load provides a stronger information on the risk of developing dnDSA than HLA antigen mismatching, particularly concerning class II HLA epitopes (22). In our study, 5 on 10 patients would have been considered at high-risk of dnDSA occurrence; among whom 3/5 indeed developed dnDSA (2 following CNI withdrawal, and one at long-term follow-up), suggesting that none of them were in fact eligible to a drug minimization strategy. Moreover, recently multiple potent blood and urinary biomarkers have emerged (23–27) which are currently part of the ongoing FDA workshop on individualized treatments in transplantation (28). Analysis of circulating blood gene signatures in stable KTR, tolerant KTR and patients with allograft rejection may also help to classify patients at-risk of developing dnDSA and BPAR (29, 30). These are complementary to the analysis of immune circulating cells, which are involved in allograft rejection (31, 32) or in the opposite process of allograft tolerance (33, 34). Also, evaluation of immunosuppressive drug metabolism can potentially identify KTR which remain at-risk of immunological complications because of insufficient drug exposure (35, 36). Whilst the pipeline for potential biomarkers of rejection is large, very few of them have currently demonstrated a significant impact in routine practice. Moreover, as all these markers were established in KTR undergoing immunosuppressive drugs, their transposition and validation for drug minimization/withdrawal need to be proven. Our series (as the one of Hricik (9)) seems to confirm the crucial role of eplet mismatch load regarding occurrence of dnDSA in patients who underwent a tacrolimus withdrawal, as 2 on 3 patients with a single DQ eplet mismatch > 11 developed dnDSA immediately following tacrolimus withdrawal.

Importantly, the risk of alloimmune reaction may not be the only factor to consider in the decision to reduce immunosuppression in KTR. Indeed, risk of nephropathy recurrence (37, 38) or adherence to treatment are important in this setting. The latter may be difficult to assess and maintain in the long-term, thus requiring a close follow-up to detect earlier potential issues, especially if maintenance therapy has been reduced (39).

Finally, some studies have elaborated dynamic prediction scores of allograft failure which can help to accurately assess the risk of failure in kidney transplant recipients (40–43). Altogether, the combination of these multiple parameters for determination of the patient immune risk may guide selection of very-low risk patients which can be eligible for a drastic minimization of immunosuppression (44, 45).

## Patient perspectives

Minimization of immunosuppression, particularly of CNI, remains a challenging objective, mainly because of the issues relating to proper selection of very low immunological risk KTR. Significant improvements have been made over the last decade in relation to evaluating the risk of immunological complications such as dnDSA and allograft rejection. Many of these at-risk markers are currently ongoing an evaluation to determine their place in routine clinical practice. Together, this knowledge may permit a significant improvement in selection of patients who may benefit from CNI minimization without increasing the risk of allograft rejection.

## Données Informatisées et VAlidées en Transplantation, DIVAT Cohort Collaborators (Medical Doctors, Surgeons, HLA Biologists)

Nantes: Gilles Blancho, Julien Branchereau, Diego Cantarovich, Anne Cesbron, Agnès Chapelet, Jacques Dantal, Anne Devis, Florent Delbos, Clément Deltombe, Lucile Figueres, Raphael Gaisne, Claire Garandeau, Magali Giral, Caroline Gourraud-Vercel, Maryvonne Hourmant, Christine Kandel-Aznar, Georges Karam, Clarisse Kerleau, Delphine Kervella, Claire Leman, Alice Leclech, Christophe Masset, Aurélie Meurette, Karine Renaudin, Simon Ville, Alexandre Walencik.

## Data availability statement

The original contributions presented in the study are included in the article/supplementary materials. Further inquiries can be directed to the corresponding authors.

## Ethics statement

The studies involving human participants were reviewed and approved by NCT01292525. The patients/participants provided their written informed consent to participate in this study.

## Author contributions

J-PS, SB, and MG elaborated the initial clinical trial. CM, JD, and MG provided data on long-term follow-up of patients. AW and FD were in charge of anti-HLA antibodies analysis. CM wrote the manuscript and all the authors participated in the revision of the initial draft. All authors contributed to the article and approved the submitted version.

## Funding

This work was supported by the European BIO-DrIM consortium (n° 305147; www.biodrim.eu) including the randomized clinical trial WEANING (NCT01292525). ANR project KTD-innov (ANR-17- RHUS-0010). Roche Pharma, Novartis, Sanofi, Chiesi and Astellas laboratories supported the DIVAT cohort. The funders were not involved in the study design, collection, analysis, interpretation of data, the writing of this article or the decision to submit it for publication.

## Conflict of interest

The authors declare that the research was conducted in the absence of any commercial or financial relationships that could be construed as a potential conflict of interest.

## Publisher’s note

All claims expressed in this article are solely those of the authors and do not necessarily represent those of their affiliated organizations, or those of the publisher, the editors and the reviewers. Any product that may be evaluated in this article, or claim that may be made by its manufacturer, is not guaranteed or endorsed by the publisher.

## Abbreviations

KTR, Kidney Transplant Recipients; CNI, Calcineurin Inhibitors; BPR, Biopsy Proven Rejection; dnDSA, *de novo* Donor Specific Antibodies; MFI, Mean Fluorescence Index; cABMR, chronic Antibody Mediated Rejection.
